# Unmasking Pernicious Anemia: A Reversible Cause of Pancytopenia Due to Severe Vitamin B12 Deficiency

**DOI:** 10.7759/cureus.87911

**Published:** 2025-07-14

**Authors:** Bola Habeb, Sandy Khair, Amber Reid

**Affiliations:** 1 Internal Medicine, Ascension Sacred Heart/Florida State University College of Medicine, Pensacola, USA; 2 Radiation Oncology, National Cancer Institute, Cairo University, Cairo, EGY

**Keywords:** anti-intrinsic factor antibodies, autoimmune gastritis, pernicious anemia, reversible cytopenias, severe pancytopenia, severe vitamin b12 deficiency

## Abstract

Vitamin B12 (cobalamin) is a vital cofactor in DNA synthesis and hematopoiesis, and its deficiency can lead to a wide spectrum of clinical manifestations, ranging from asymptomatic macrocytosis to severe hematologic and neurologic complications. While megaloblastic anemia is the most common presentation, prolonged or profound deficiency can result in pancytopenia due to ineffective hematopoiesis and intramedullary cell death. This can mimic more serious bone marrow disorders, including leukemia or aplastic anemia, potentially leading to invasive diagnostic procedures if the deficiency is not promptly recognized. Pernicious anemia is the most common cause of vitamin B12 deficiency in developed countries. It is an autoimmune condition characterized by intrinsic factor deficiency due to antibodies targeting either intrinsic factor itself or gastric parietal cells. It often presents insidiously and is frequently diagnosed late, typically after complications such as pancytopenia or irreversible neurologic damage have developed. Timely identification and treatment with parenteral vitamin B12 can lead to rapid hematologic recovery and prevention of permanent neurologic injury, highlighting the importance of considering pernicious anemia in the differential diagnosis of pancytopenia.

## Introduction

Vitamin B12 (cobalamin) is an essential cofactor in DNA synthesis, particularly in rapidly dividing cells such as hematopoietic precursors. Deficiency of this micronutrient impairs nuclear maturation and cell division, resulting in megaloblastic changes within the bone marrow and peripheral cytopenias. Although macrocytic anemia is the most common hematologic manifestation, severe or prolonged deficiency can progress to pancytopenia. This rare but potentially life-threatening presentation occurs in approximately 5% to 10% of cases and can mimic hematologic malignancies or marrow failure syndromes [1-3].

The most common causes of vitamin B12 deficiency include dietary insufficiency (particularly in vegans), malabsorption syndromes, and pernicious anemia. Pernicious anemia is an autoimmune disorder characterized by the presence of anti-intrinsic factor or anti-parietal cell antibodies that impair cobalamin absorption in the terminal ileum. It remains a leading cause of B12 deficiency in developed countries, especially among older adults [4-6]. Clinical manifestations range from nonspecific symptoms such as fatigue and glossitis to neuropsychiatric disturbances, including cognitive impairment, depression, irritability, and psychosis, as well as hematologic abnormalities like anemia, leukopenia, thrombocytopenia, and, in rare instances, pancytopenia [7].

Timely recognition and treatment of vitamin B12 deficiency are critical, as hematologic abnormalities are reversible with cobalamin supplementation, and early therapy may prevent irreversible neurologic complications. This case report describes a patient presenting with pancytopenia as the initial manifestation of previously undiagnosed pernicious anemia, highlighting the importance of considering vitamin B12 deficiency in the differential diagnosis of pancytopenia to ensure prompt and effective treatment.

## Case presentation

A 75-year-old male with a medical history of hypertension, hyperlipidemia, and a prior transient ischemic attack was admitted for further evaluation of anemia identified during routine outpatient laboratory testing. He reported progressively worsening fatigue, generalized weakness, and exertional dyspnea over the past six months, with notable symptom exacerbation in the last two months. Additionally, he endorsed a poor appetite and an unintentional weight loss of approximately 30 pounds during this time. He denied following a vegan diet, as well as any overt gastrointestinal bleeding, melena, or chronic use of nonsteroidal anti-inflammatory drugs (NSAIDs). There was no history of prior blood transfusions. His most recent screening colonoscopy, performed five years ago, was unremarkable, and he had never undergone esophagogastroduodenoscopy (EGD). He also denied tobacco use, alcohol consumption, or illicit drug use.

His home medications included losartan-hydrochlorothiazide 100 mg/25 mg once daily, nifedipine 30 mg extended-release tablet once daily, and simvastatin 40 mg once daily. He denied the use of any additional over-the-counter medications, including proton pump inhibitors, H2-receptor antagonists, and herbal supplements.

Clinical findings

On physical examination, the patient was afebrile with a temperature of 36.8°C, blood pressure of 134/84 mmHg, heart rate of 103 beats per minute, respiratory rate of 18 breaths per minute, and oxygen saturation of 97% on room air. He was alert and oriented to person, place, and time, with no motor or sensory deficits. Scleral pallor was noted. The abdominal examination revealed a soft, non-tender, and non-distended abdomen with active bowel sounds. Cardiovascular examination demonstrated a regular rate and rhythm without murmurs. Pulmonary auscultation revealed clear breath sounds bilaterally, with no wheezes, rales, or rhonchi. No petechiae or ecchymosis were observed on skin examination.

Diagnostic assessment

Laboratory results on admission are demonstrated in Table 1.

**Table 1:** Laboratory data obtained on admission. *Abnormal laboratory values. MCV: mean corpuscular volume; BUN: blood urea nitrogen; AST: aspartate aminotransferase; ALT: alanine aminotransferase; INR: international normalized ratio; TSH: thyroid stimulating hormone; A1C: glycated hemoglobin

Parameters	Patient's values on admission	Reference range, adults
Hemoglobin (g/dL)	3.6*	12.0-15.5
Hematocrit (%)	10.2*	34.9-44.5
MCV (fL)	106.3	81-98
White cell count (per mm^3^)	1,800*	3,500-10,500
Platelet count (per mm^3^)	67,000*	150,000-450,000
Sodium (mEq/dL)	141	135-145
Potassium (mEq/dL)	4.7	3.5-5.1
Bicarbonate (mEq/dL)	21	22-29
BUN (mg/dL)	21*	12-21
Creatinine (mg/dL)	1.69*	0.72-1.25
ALT (units/L)	51	11-55
AST (units/L)	80*	5-34
Alkaline phosphatase (U/L)	56	40-150
Total bilirubin (mg/dL)	2.6*	0.2-1.2
Direct bilirubin (mg/dL)	0.7*	0.1-0.5
Indirect bilirubin (mg/dL)	1.9*	0.0-1.2
INR	1.3	Critical high >4
TSH (mclU/mL)	0.74	0.35-4.9
A1C	6.1	≤6.5

Urinalysis demonstrated yellow-colored urine with +3 leukocyte esterase, 30 mg/dL protein, 2 mg/dL urobilinogen, >100 white blood cells, and microscopic hematuria with 64 red blood cells per high-power field (HPF). Based on the initial laboratory tests, further workup was obtained and demonstrated in Table 2.

**Table 2:** Additional laboratory tests. *Abnormal laboratory values. TIBC: total iron binding capacity; LDH: lactate dehydrogenase; DAT: direct antiglobulin test; DAT ANTI-C3BD: direct antiglobulin test for complement; Hep A IgM: hepatitis A virus immunoglobulin M; Hep Bs Ag: hepatitis B virus surface antigen; Hep C Ab: hepatitis C virus antibody; Hep B core IgM: hepatitis B virus core antibody, immunoglobulin M; HIV Ag, Ab: human immunodeficiency virus antigen, antibody; EBV Ab panel: Epstein-Barr virus antibody panel; ANA: antinuclear antibody; anti-Ds DNA: anti-double stranded DNA antibody; Ig A: immunoglobulin A; Deam Gliad IgA Abs: deamidated gliadin peptide immunoglobulin A antibodies; Deam Gliad IgG Abs: deamidated gliadin peptide immunoglobulin G antibodies; tTG IgA: tissue transglutaminase IgA antibodies; tTG IgG: tissue transglutaminase IgG antibodies; CK: creatine kinase

Parameters	Patient's values on admission	Reference range, adults
	Anemia workup	
B12 (pg/mL)	<150*	213-816
Folate (ng/mL)	12.3	7-31.4
Iron (mcg/dL)	231*	65-175
TIBC (mcg/dL)	Unable to calculate	280-400
Iron saturation, %	Unable to calculate	11-46
Ferritin (ng/mL)	1,041*	21.8-274
LDH (IU/L)	3,325*	125-220
Haptoglobin (mg/dL)	<8	40-268
Reticulocytes, %	1.6%	0.3-2.4
DAT - Anti-IgG Coombs serum (units)	Positive	-
DAT ANTI-C3BD (units)	Positive	-
	Infectious workup	
Hep A IgM	Nonreactive	-
Hep Bs Ag	Nonreactive	-
Hep C Ab	Nonreactive	-
Hep B core IgM	Nonreactive	-
HIV Ag, Ab combo screen	Nonreactive	-
EBV Ab panel	Negative	-
	Autoimmune workup	
ANA	Negative	-
Anti-Ds DNA (IU/mL)	1.1	<9.9
C3 complement (mg/dL)	62	90-180
C4 complement (mg/dL)	36	15-57
Ig A (mg/dL)	105	61-437
Intrinsic factor antibody (AU/mL)	19.8*	0-1.1
Deam Gliad IgA Abs	3	0-19
Deam Gliad IgG Abs	2	0-19
tTG IgA (units/mL)	2	0-5
tTG IgG (units/mL)	3	0-5
	Miscellaneous	
Total homocysteine (mcmol/L)	69.24*	5.08-15.39
Methylmalonic acid (nmol/L)	30,943	0-378
CPK (IU/L)	26	30-200

**Figure 1:**
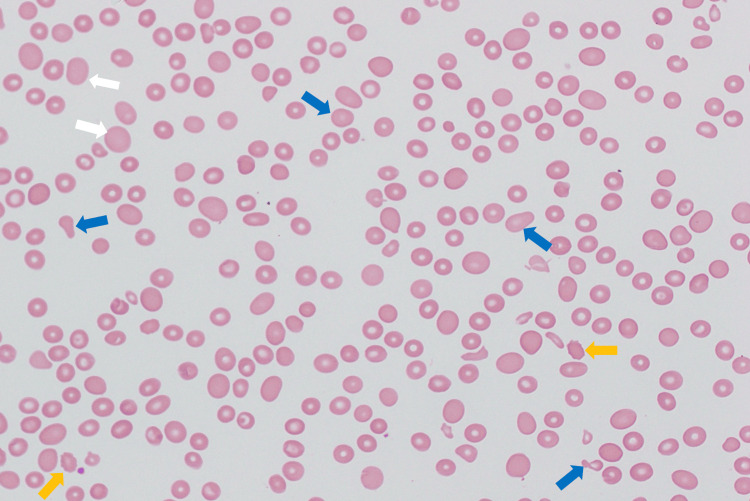
Peripheral blood smear. White arrows represent macrocytosis. Blue arrows represent anisopoikilocytosis. Yellow arrows represent schistocytes.

A peripheral blood smear was obtained, revealing pancytopenia with macrocytosis and anisopoikilocytosis, including the presence of schistocytes (Figure 1).

The patient’s constellation of symptoms, including fatigue, weakness, and dyspnea, combined with pancytopenia, severe macrocytic anemia, profound vitamin B12 deficiency, evidence of intramedullary hemolysis, and positive intrinsic factor antibodies, is highly indicative of pernicious anemia as the underlying etiology.

Treatment

The patient received a total of four units of packed red blood cells (PRBCs) during hospitalization and was initiated on intramuscular vitamin B12 at a dose of 1,000 mcg daily for one week. He was advised to continue B12 injections weekly for four weeks, followed by monthly maintenance therapy. The patient received dietary counseling for severe vitamin B12 deficiency, with guidance to increase intake of B12-rich foods such as meat, fish, dairy products, and fortified cereals. Emphasis was placed on the importance of adherence to prescribed supplementation, as dietary sources alone are typically insufficient to correct severe deficiency. Potential causes of malabsorption, including gastrointestinal conditions and medication use, were discussed. Ongoing monitoring of B12 levels and clinical response was recommended.

Although the urinalysis was suggestive of a urinary tract infection, the patient remained asymptomatic; therefore, antibiotics were not initiated, consistent with guidelines for the management of asymptomatic bacteriuria.

At his one-week follow-up after discharge, laboratory studies demonstrated marked improvement: hemoglobin had increased to 10.4 g/dL, white blood cell count was 7,300/µL, and platelet count had normalized to 420,000/µL. Additionally, lactate dehydrogenase (LDH) levels declined from 3,325 IU/L to 1,035 IU/L, and haptoglobin increased from <8 mg/dL to 121 mg/dL, indicating resolution of intramedullary hemolysis.

## Discussion

Vitamin B12 is primarily obtained from animal-based dietary sources such as meat, poultry, fish, eggs, and dairy products, making individuals who follow strict vegan diets particularly susceptible to deficiency if not appropriately supplemented [1]. Beyond inadequate intake, vitamin B12 deficiency commonly arises from impaired absorption due to gastrointestinal or systemic conditions. In developed countries, the most prevalent cause is pernicious anemia, an autoimmune condition characterized by the destruction of gastric parietal cells or the presence of intrinsic factor antibodies, both of which disrupt B12 absorption in the terminal ileum [4]. Other etiologies include malabsorptive disorders such as celiac disease, Crohn’s disease, chronic atrophic gastritis, and small intestinal bacterial overgrowth, as well as post-surgical states involving gastrectomy or ileal resection [8]. Additionally, long-term use of medications such as proton pump inhibitors, H2-receptor antagonists, and metformin has been associated with reduced B12 absorption [9,10]. Identifying the underlying cause is essential to guide targeted therapy and prevent recurrence.

Vitamin B12 deficiency can present with a wide array of clinical manifestations affecting the hematologic, neurologic, and psychiatric systems. Hematologically, it typically presents with megaloblastic anemia, but in more advanced stages, it may progress to pancytopenia due to ineffective hematopoiesis and intramedullary apoptosis. Laboratory findings may include elevated LDH and indirect hyperbilirubinemia, reflecting increased cell turnover and ineffective erythropoiesis [3,4]. Neurologic complications include peripheral neuropathy, loss of proprioception and vibration sense, gait ataxia, and subacute combined degeneration of the spinal cord, often manifesting as spastic paraparesis [11,12]. Psychiatric and cognitive symptoms, such as depression, irritability, memory loss, psychosis, and even dementia-like syndromes, are also well documented, especially among older adults [13]. Importantly, neurologic deficits may occur independently of hematologic abnormalities and can become irreversible if not promptly treated [11].

While macrocytic anemia is a hallmark of B12 deficiency, the diagnosis may be overlooked in patients who present with leukopenia or thrombocytopenia without prominent neurologic symptoms. Pancytopenia occurs in approximately 5% to 10% of B12-deficient cases, making it an uncommon but clinically significant finding that can mimic bone marrow failure syndromes or hematologic malignancies [2]. In such presentations, failure to recognize B12 deficiency may result in extensive and unnecessary investigations, including bone marrow biopsy, delay in treatment, and potentially avoidable complications.

Among all causes of B12 deficiency, pernicious anemia is the most common in older adults in developed countries. The diagnosis is supported by low serum B12 levels, elevated methylmalonic acid and homocysteine, and the presence of intrinsic factor antibodies, which are highly specific for this autoimmune condition [5,6]. Although bone marrow biopsy is not routinely indicated, when performed, it typically reveals a hypercellular marrow with nuclear-cytoplasmic asynchrony and megaloblastic changes consistent with ineffective erythropoiesis [14].

In our case, the patient’s clinical presentation, marked by progressive fatigue, generalized weakness, and exertional dyspnea, along with laboratory findings of severe macrocytic anemia, leukopenia, thrombocytopenia, and profound vitamin B12 deficiency, strongly pointed toward a hematologic disorder. Supporting evidence of intramedullary hemolysis included elevated LDH, undetectable haptoglobin, a positive direct Coombs test, and the presence of schistocytes on peripheral smear. The detection of intrinsic factor antibodies further confirmed the diagnosis of pernicious anemia as the underlying cause of pancytopenia. Contributing etiologies such as celiac disease, diabetes mellitus, thyroid dysfunction, autoimmune hemolytic anemia, and rhabdomyolysis were effectively ruled out. This was supported by negative celiac antibodies, normal hemoglobin A1c, normal TSH, normal complement levels, negative ANA, and an unremarkable infectious workup including hepatitis, HIV, and EBV. Additionally, despite the presence of hematuria on urinalysis and a history of statin use, a normal creatine kinase level made rhabdomyolysis unlikely.

Iron studies demonstrated a pattern consistent with functional iron overload, a phenomenon frequently observed in cases of ineffective erythropoiesis due to B12 deficiency. In this context, the destruction of immature erythroid precursors within the bone marrow releases intracellular iron into the circulation. This leads to characteristic laboratory findings, including elevated serum iron, increased ferritin levels, high transferrin saturation, and low to normal total iron-binding capacity (TIBC). Although these values may mimic true iron overload, the condition is functional and typically resolves with appropriate vitamin B12 replacement therapy.

Guidelines recommend parenteral vitamin B12 replacement for patients with symptomatic deficiency or malabsorption, as this ensures reliable absorption and rapid hematologic recovery [4,15]. The standard regimen involves intramuscular injections of 1000 mcg daily or every other day for one to two weeks, followed by weekly injections until blood counts normalize, then monthly maintenance therapy for life, particularly in cases of irreversible malabsorption or pernicious anemia [16]. A reticulocyte response is typically seen within five to seven days, with progressive improvement in anemia, leukopenia, and thrombocytopenia over several weeks. Regular monitoring, including CBC and vitamin B12 levels every 6-12 months, is crucial to ensure hematologic stability and detect early relapse. Patients should be educated on the need for lifelong therapy and encouraged to report any recurrence of symptoms such as fatigue, neuropathy, or cognitive changes [17].

Clinical studies and case reports consistently demonstrate that over 90% of patients with pancytopenia due to B12 deficiency achieve complete hematologic recovery following appropriate treatment [3]. However, delays in diagnosis can lead to persistent or irreversible neurologic deficits, reinforcing the need for heightened clinical awareness and early intervention.

This case contributes to the existing literature by illustrating a classic yet underrecognized presentation of pernicious anemia manifesting as pancytopenia. It highlights the importance of including vitamin B12 deficiency in the differential diagnosis of unexplained cytopenias, particularly in patients with macrocytosis, elevated LDH, or known risk factors for malabsorption. Timely recognition and appropriate treatment can prevent unnecessary procedures, reverse hematologic abnormalities, and avert irreversible neurologic complications.

## Conclusions

Vitamin B12 deficiency, particularly due to pernicious anemia, should be considered in the differential diagnosis of pancytopenia, especially when accompanied by macrocytosis and elevated LDH. Although pancytopenia is an uncommon manifestation, it represents an advanced stage of deficiency with significant clinical implications. Early identification and treatment with parenteral cobalamin can lead to complete hematologic recovery and prevent permanent neurologic damage. A thorough diagnostic approach, including assessment for intrinsic factor antibodies, can facilitate accurate diagnosis and timely management, avoiding unnecessary interventions and improving patient outcomes.
